# *In silico* and pharmacological evaluation of GPR65 as a cancer immunotherapy target regulating T-cell functions

**DOI:** 10.3389/fimmu.2024.1483258

**Published:** 2024-10-17

**Authors:** Shamin Li, Fabien Melchiore, Chahrazade Kantari-Mimoun, Aurore Mouton, Samantha Knockaert, Wendy Philippon, Benjamin Chanrion, Christophe Bourgeois, Céline Lefebvre, Jamila Elhmouzi-Younes, Véronique Blanc, Fernando Ramon Olayo, Bruno Laugel

**Affiliations:** ^1^ Institut de Recherches Servier, Paris-Saclay R&D Center, Gif-sur-Yvette, France; ^2^ Institut de Recherches Internationales Servier, Paris-Saclay R&D Center, Gif-sur-Yvette, France

**Keywords:** acidosis, immunosuppression, immunotherapy, GPCRs, Gpr65, T-cells, solid tumours, TDAG8

## Abstract

The success of cancer immunotherapies such as immune checkpoint inhibitors, CAR T-cells and immune cell engagers have provided clinicians with tools to bypass some of the limitations of cancer immunity. However, numerous tumour factors curtail the immune response against cancer and limit the efficiency of immuno-oncology (IO) therapies. Acidification of the extra-cellular tumour environment consecutive to aberrant cancer cell metabolism is a well-known promoter of oncogenic processes that also acts as an immune regulator. Yet, the suppressive mechanisms of low extra-cellular pH on anti-cancer immunity remain poorly understood. Recent reports have suggested that GPR65, a Gαs-coupled proton-sensing GPCR broadly expressed in the immune system, may act as an immune suppressant detrimental to anti-tumour immunity. So far, the immuno-regulatory properties of GPR65 in acidic milieux have mostly been documented in macrophages and myeloid cells. Our computational evaluation of GPR65’s transcriptomic expression profile and potential as an IO target using public datasets prompted us to further investigate its functions in human T-cells. To this end, we identified and validated GPR65 small molecule inhibitors active in *in vitro* cellular assays and we showed that GPR65 inhibition promoted the killing capacity of antigen-specific human T-cells. Our results broaden the scope of GPR65 as an IO target by suggesting that its inhibition may enhance T-cell anti-tumour activity and provide useful pharmacological tools to further investigate the therapeutic potential of GPR65 inhibition.

## Introduction

Cancer immunotherapies have changed the landscape of clinical practice in oncology by providing therapeutic modalities with novel mechanisms of action that show durable clinical benefits and favorable toxicity profiles. Immune checkpoint (ICP) blockade with monoclonal antibodies is probably the most remarkable achievement of this therapeutic approach, showing success in several indications and moving in earlier treatment lines. However, despite a wealth of promising ICP targets identified in the preclinic, few have shown efficacy in patients. To this day, only the PD-1/PD-L1 and CTLA-4 pathways have reached the market based on outstanding monotherapy activities, highlighting the need to identify other tractable targets with therapeutic potential as single agents or in combination with other IO assets.

The common denominator of successful cancer immunotherapies (ICP blockers, cellular therapies and immune-cell engagers) appears to be their ability to harness the anti-tumour activity of T-cells (1). T-cells are crucial levers instructing the immune system to efficiently combat tumours. Like all immune cells, they are sensitive to the inhibitory activity of multiple cues emanating from cancer cells and from the tumour micro-environment (TME) (1). Beyond cell surface ICPs such as PD-1 or LAG-3, myriads of soluble hormones, cytokines or metabolites can act to support tumourigenesis either by directly promoting cancer survival and growth or by suppressing anti-tumour immunity (2, 3). Extra-cellular milieu acidification is a well-known by-product of rapidly proliferating cancer cells that rely on aerobic glycolysis (4). This aberrant metabolic process also reinforces oncogenesis by activating pro-survival/proliferation pathways (5, 6). In addition, low extra-cellular pH in the TME can also directly inhibit immune cells and curtails immune cells activation, including tumour infiltrating T-cells (TILs), through local increases in lactate and H+ concentrations (7, 8).

GPR65, also referred as TDAG8 was identified as a proton-sensing G protein coupled receptor (GPCR) widely expressed in the immune system and likely involved in acidosis-mediated immune suppression (9–11). GPR65 is coupled to Gαs proteins and triggers the cAMP/PKA second messenger pathway, known to exert potent inhibitory effects in multiple cell types of the immune system (12, 13) in a manner similar to well-characterized metabolite-sensing GPCRs such as the prostaglandin E2 receptors EP2/EP4 and the adenosine receptors ADORA2A and 2B (14–20). At steady-state, GPR65 expressed on both immune cells and intestinal epithelial cells seems important to maintain immune homeostasis in the gastro-intestinal (GI) tract by regulating the commensal and pathogenic flora (21–23). GPR65-dependent promotion of phagocytosis and anti-microbial peptide secretion in the intestinal lumen have been involved in its GI homeostatic functions. In addition, proton sensing by GPR65 skews the functions of macrophages towards an anti-inflammatory phenotype, likely contributing to immune homeostasis in other tissues (24, 25). Numerous genome wide association studies (GWAS) linking GPR65 polymorphism to increased incidence of inflammatory bowel disease (IBD) and other autoimmune/autoinflammatory diseases (26–30) highlight the importance of GPR65’s regulatory functions on the immune system. The single nucleotide polymorphism (SNP) rs3742704 linked to autoimmunity was reported in 9.2% of individuals of European descent (31) and in 20.6% of East Asian populations (32). rs3742704 encodes an Isoleucine to Leucine substitution at position 231 (I231L) of the GPR65 protein, which results in reduced cAMP signaling in response to low pH compared to the reference allele. Remarkably, this partial GPR65 loss of function leads to dysbiosis in the gut and compromised intestinal barrier functions through dysregulated lysosomal homeostasis, thereby providing a mechanistic link with susceptibility to IBD (21). Similarly, impaired GPR65 functions altered endo-lysosomal trafficking and proteolysis in myeloid cells, resulting in defective bacterial clearance but also in increased antigen presentation to CD4+ T-cells (33). In addition, GPR65 I231L knock-in mice showed exacerbated pro-inflammatory dendritic cells (DCs) functions at low pH (33). These results highlight the immune-stimulatory and pro-inflammatory effects of diminished GPR65 functions, also suggesting that pharmacological targeting of this receptor may provide a new approach to modulate immune functions in low pH environments. Several reports have shown that GPR65 deletion or altered signalling affected mouse T-cell polarization and functions, notably by preventing Th17 differentiation (25, 33, 34), yet, to date, little is known about the T-cell-intrinsic role of GPR65. Mechanistic insights into these observations are lacking and it is unclear whether these effects are T-cell-intrinsic or indirect, through interactions with antigen-presenting cells. To the best of our knowledge, the role of GPR65 in T-cell activation was only addressed in a single report where a synthetic GPR65 agonist inhibited IL-2 secretion in mouse T-cells stimulated with anti-CD3/CD28 antibodies (35).

Considering that extra-cellular milieu acidification consequent to a highly glycolytic cancer cell metabolic state is a common feature of solid tumours, we were interested in the potential immune suppressive role of GPR65 in the context of T-cell anti-tumour immunity. We report *in silico* investigations suggesting that GPR65 inhibition could be beneficial for cancer immunotherapy and that human T-cell functions are downmodulated by GPR65. Moreover, we provide evidence that antagonizing GPR65 with small molecule inhibitors improves the ability of T-cells to inhibit the growth of tumour cells *in vitro* and augments cytokine secretion by T-cells. Our results shed light on the immunomodulatory role of GPR65 and suggest that therapeutic intervention aimed at inhibiting GPR65 could improve anti-tumour immunity through the action of several immune cell types, including T-cells, a population with demonstrated anti-cancer potential in the context of IO therapeutic interventions.

## Materials and methods

### TCGA patient survival stratification according to GPR65 genotype and co-variate analyses

Preprocessing of genotypes data: TCGA patients genotypes from Whole Exome Sequencing (WES) data have been retrieved from the GDC portal (accession to phs000178.v11.p8 was granted for the project through dbgap). Filtered variant calls of 10,389 final passed-QC samples were then combined and merged. All variants were annotated using the SNPEFF tool (v5.1d) and COSMIC database (v99). GPR65 genotypes were then classified in three groups (WT/HET/HOM) or two groups (nonHOM [=HET & WT]/HOM) where WT refers to homozygosity for the reference allele, HOM to homozygosity for the rs3742704 risk allele and HET to the presence of both alleles.

Preprocessing of sample annotations: TCGA patient and sample annotations have been retrieved from GDC portal. TCGA patients’ overall survival (OS) data were retrieved from (36). Stages of cancer have been re-coded in a ‘Pooled Stage’ variable combining ‘Clinical Stage’ and ‘Pathologic Stage’ TCGA annotations; stages I and II were coded ‘Early stage’ whereas stages III and IV were coded ‘Late stage’. Immune subtypes or clusters were retrieved from Thorsson et al. (37).

Selection of samples and indications: only tumour samples with associated patient OS and genotypes from WES data were selected for this analysis.

Survival analysis: Kaplan-Meier curves and Log Rank ratio test have been computed using Surv(), survit() and ggsurvplot() functions from ‘survival’ (v3.5-5) and ‘survminer’ (v0.4.9) R libraries. Cox model and Hazard ratio analyses have been done using Surv() and coxph() functions from the same R packages, under R v4.3.1. For analyses using Cox model, indications with less than 2 samples in one GPR65 rs3742704 genotype group were filtered out.

### Public single-cell RNA sequencing (scRNAseq) data processing and analyses

We deciphered the pan-cancer expression pattern of GPR65 gene at single-cell level using TISCH2 resource (Han et al., 2023). Datasets annotations and GPR65 gene expression table have been downloaded from TISCH website. Cell types have been grouped in three compartments: Immune cells, Malignant cells and Stromal cells. Only TME datasets composed of at least annotated malignant cells and TILs were selected for this analysis. ComplexHeatmap (v2.16.0) R package was used to plot GPR65 expression levels by cell type across annotated datasets.

Cell populations from BioTuring database: GPR65 gene expression level in the different cell populations of interest was evaluated using BioTuring Talk2Data tool and Human Single-cell RNA sequencing database v4.0 (Bioturing Inc.).

### Small molecule pharmacological modulators of GPR65

The GPR65 agonists BTB09089 3-[(2,4-dichlorobenzyl)thio]-1,6-dimethyl-5,6-dihydro-1H-pyridazino [4,5-e][1,3,4]thiadiazin-5-one (38) and ZINC13684400 N-[4-(3,4-dimethoxyphenyl)-1,3-thiazol-2-yl]-2-(1H-1,2,4-triazol-5-ylsulfanyl) acetamide (39) were purchased from Aurora Fine Chemicals LTD (Graz, Austria) and MedChemExpress LLC (Monmouth Junction, USA), respectively. The GPR65 antagonists referred to as SD2571, SD2593, SD2594 and SD2758 used in this report are described in **Supplementary Figure 5** and were synthesized by WuXi Apptech (Shanghai, China) based on structures reported in the patent international application PCT/GB2021/051397.

### Lentivirus production

A 3rd generation packaging system was used, including 4 vectors for additional safety: the lentivector of interest encoding the genes of interest (TCR or GPR65), the packaging (pRSV-REV), envelop (pVSV-G) and accessory plasmids (pMDLg/pRRE). The lentivectors encoding TCRs specific for HLA-A2/NY-ESO1_157-165_ (40), HLA-A2/MAGE-A4_230-239_ (41) or the rCD2-GPR65 tandem construct (**Supplementary Figure 3A**) were added to the packaging mix containing 1 mg/ml of pRSV-REV, 1 mg/ml of pMDLg/pRRE and 0.5 mg/ml of pVSV-G in sterile milliQ water. 500ul of total packaging plasmid mix were aliquoted per 1.5ml Eppendorf tube. HEK293T cells were seeded in a T-150 culture flask in DMEM supplemented with 10% FBS, Pen/Strep and 10mM HEPES 3 days prior to transfection to reach around 50% confluence. Culture medium was renewed just before transfection at day 0 and the transfection solution containing 100ul of TurboFect (ThermoFischer, #R0534) transfection agent, 67.5ul of the packaging plasmid mix, 15ug of the transfer lentivector in 3ml of culture medium (DMEM + 10mM HEPES) was carefully added. The flask was gently swirled and incubated at 37°C, 5% CO2. Supernatants were collected 48h post transfection, cell and debris were removed by centrifugation at 1000xg for 5min at 4°C, and supernatants were further filtered using 0.45 µM nylon syringe filters. Viral particles were concentrated by ultracentrifugation for 4h at 10000xg at 4°C without brake, resuspended in cold cell culture medium (RPMI-1640 supplemented with 10% FBS, 1% Penicillin/Streptavidin and 1% HEPES medium) and snap frozen on dry ice.

### TCR T-cell production and phenotypic characterization

Buffy coats were obtained from healthy donors at EFS (Etablissement Français du Sang) with patients consent. A ficoll preparation was performed to isolate peripheral blood mononuclear cells (PBMCs) using Lymphoprep (Stemcell #07851). CD14 depletion was performed to enrich peripheral blood T (PBT) cells using human CD14 Microbeads (Miltenyi #130-050-201). TCR-T cells were produced from freshly prepared PBT cells or a frozen stock, in G-Rex^®^6 Well Plate system (Wilson Wolf, #80240M). Briefly, PBT cells were resuspended on day 0 at a density of 1E6 cells per ml of X-Vivo15 (Lonza, #02-060Q), with 10% inactivated Human serum AB (BIOIVT, #GEM-100-512-H) + 1% Pen/Strep supplemented with T-cell Transact (Miltenyi, #130-111-160) at 1/55 dilution and IL-2 (R&D Systems, #202-IL) at 25ng/ml and incubated overnight at 37°C, 5% CO2. On day 1, transduction was performed by adding the frozen NY-ESO1 or MAGE-A4 TCR lentivirus + Synperonic F108 at 1mg/ml (SigmaAldrich, #07579-250G-F) and IL-2 at 25ng/ml. Culture medium was renewed 2 days later, and 25ng/ml of IL-2 was added at D1, D5, D7, D9 and D12. On both day 5 and 14, TCR expression and cell phenotype were characterized by Flow cytometry (**Supplementary Figure 6A**); followed by TCR-T cell tumour cell killing assay or cell freezing.

### Generation of A375 cells overexpressing GPR65

A375 cells (ATCC, #CRL-1619) stably overexpressing GPR65 were cultured in DMEM GlutaMAX supplemented with 10% inactivated FBS, and 1% Pen/Strep. A375 cells were transduced with lentivectors expressing GPR65 in tandem with the rat CD2 reporter gene (**Supplementary Figure 3A**) at dilution 1/4. GPR65 expression was inferred from rCD2 staining and positive cells were purified by magnetic cell sorting after staining with anti-FITC MicroBeads (Miltenyi, #130-048-701) according to the manufacturer's protocol. The purified populations expressing rCD2 at a frequency of at least 80% (**Supplementary Figure 3B**). Cells were maintained at 37°C, 5% CO2.

### TCR-T cell antigen-specific tumour cell killing assay

A375 cells transduced with Incucyte^®^ Nuclight Red Lentivirus (EF1a, Puro) (Sartorius, #4476)) were cultured in DMEM GlutaMAX supplemented with 10% inactivated FBS, 1% Pen/Strep and 1% HEPES medium. Cells were detached using 2 ml of Enzyme TrypLE Express (Gibco, #10043382), washed and resuspended in a 96-well plate at 5000 cells/well. Compounds of interest were added at the corresponding concentrations at 50µl/well, followed by TCR-T cells at two selected E.T.ratios, at 50µl/well. The TCR-T cell killing capacity of the A375 tumour cells is assessed beforehand to ensure the right E.T. ratio to use. The tumour growth is then assessed over time through the Incucyte System S3 (Sartorius) using the total red nucleus area (µm^2^/well) with objective 10xWhen indicated, restimulation is performed by adding compounds of interest in the culture +/- A375 Nuclight Red cells, and supernatant is collected for subsequent cytokine analysis. Each condition was tested in duplicates or triplicates in 8 donors from 3 independent experiments. TCR-T cell antigen-specific tumour cell killing is inversely correlated to the area under the curve (AUC) measurement, with low value of AUC corresponding to significant killing of targets cells. Accordingly, the killing index was calculated as follows: ((AUC of untreated A375-AUC of treated A375)/AUC of control A375)*100).

### TCR T-cell activation assay by flow cytometry

Label-free A375 cells were cultured with TCR transgenic T cells as stated above. A single E.T. ratio was selected based on the Incucyte cell killing data, and cells were collected after 3 days of culture for flow cytometry analysis. Briefly, cells were washed using PBS, resuspended in Zombie IR Live/Dead dye (AAT bioquest, iFluor 860 maleimide #1480) at 1/1000 dilution and incubated at room temperature for 10min in the dark. Cells were washed with PBS without calcium or magnesium, 0.5%FBS, 2mM EDTA (PBS+) and stained with surface antibodies (**Supplementary Table 2**) for 20min at 4°C in the dark. Cells were washed with PBS+, fixed and permeabilized with the Foxp3/Transcription Factor Staining Buffer set (eBioscience, #00-5523-00) following the manufacturer’s instructions. After an incubation of 30min at room temperature in the dark, cells were washed using the permeabilization buffer and resuspended with intranuclear markers (**Supplementary Table 2**). After 30min of incubation at room temperature in the dark, cells were washed using the permeabilization buffer, resuspended in PBS + 0.5% FBS and analyzed on Cytoflex LX (Beckman Coulter).

### Cytokine release assays

Supernatants from co-cultures described above were analyzed using the Human CD8 T cell Multiplex Assay (Merck,#HCD8MAG-15K-13). Samples were prepared according to the manufacturer’s instructions and analyzed on the Luminex (Biorad).

### cAMP HFRET measurement assay

RPMI in powder form was chosen as the assay medium because it did not contain any buffer system, such as HEPES or NaHCO3, with pH regulation achieved by gaseous CO2 in incubators. The powder was resuspended at 10g/L and referred to as the ‘non-buffered medium.’ Buffered media at various pH levels were prepared using this non-buffered medium by adding a non-zwitterionic buffer (20mM final concentration). The choice of buffer depended on the targeted pH. HEPES was used to achieve a pH of 7 and 7.5, while EPPS was employed to attain a pH of 8. Once added, the pH of the buffered medium was measured with a pH meter and adjusted with HCl or NaOH, with only a few drops needed for pH adjustment.

Control or GPR65-transduced A375 cells (**Supplementary Figures 3A, B**) were harvested and resuspended in a pH 8-buffered medium to ‘de-stimulate’ the GPR65 receptor for 30 minutes. Then, cells were centrifuged and resuspended in a non-buffered medium at the appropriate concentration. 3 µl of cells were dispensed (equivalent to 400 cells/well) into 1536 Assay Ready Plates (ARP) for compound treatment. Following a 30-minute incubation at 37°C without CO2, cells were stimulated by adding 3 µl of buffered medium mixed with IBMX (500μM final) for an additional 30 minutes at 37°C without CO2. HTRF kit reagents were dispensed. cAMP Gs dynamic kit from Revvity was used. Reagents were prepared as described by Cisbio. 1μL of HTRF cAMP Gs Dynamic d2 and 1μL of HTRF cAMP Gs Dynamic EU working solutions were dispensed. The plates were incubated at room temperature in the dark. Then, Fluorescence was measured on the PHERAstar reader. Time-gated fluorescence signals were recorded at 665 nm using a 337-nm excitation wavelength, 60-µs delay, and 400-µs integration times for channel A, and at 620 nm using a 337-nm excitation wavelength, 60-µs delay, and 400-µs integration times for channel B. For data analysis, HTRF ratio (Channel B/Channel A) was converted into cAMP back-calculated concentrations using cAMP standard in ARP. In this condition, only the non-buffered medium was dispensed instead of cells. The pH of the secondary dispensed medium did not impact the cAMP standard curve in the range of tested pH.

## Results

### GPR65 risk allele rs3742704 homozygosity correlates with improved survival in cancer patients

**Figure 1:**
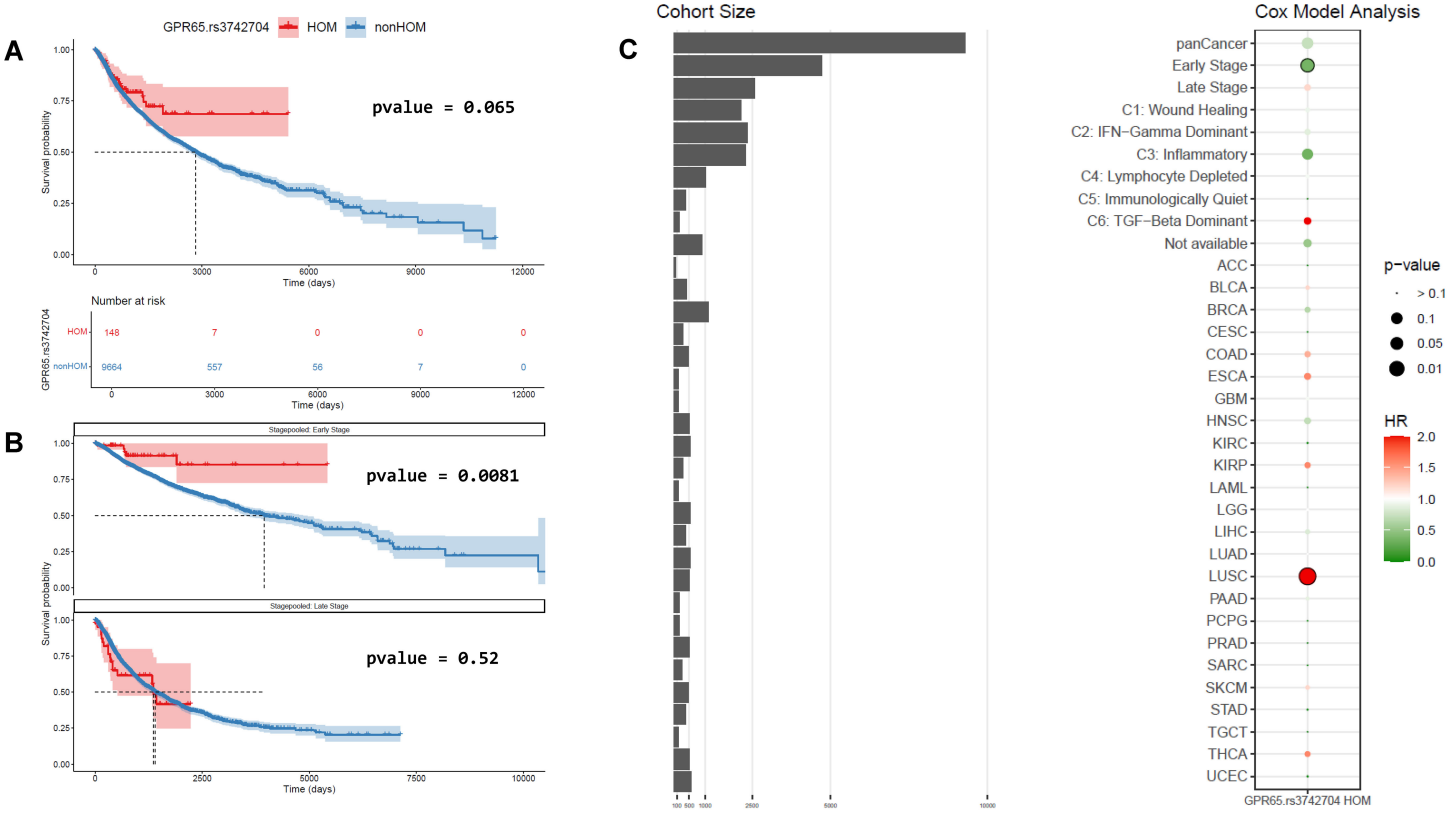
TCGA pan-cancer survival analysis regarding GPR65 genotype. **(A)** Kaplan-Meier curves and logrank test between rs3742704 homozygous and non-homozygous patients in the entire TCGA database. **(B)** Kaplan-Meier curves and logrank test between rs3742704 homozygous and non-homozygous patients in early or late stages. **(C)** Cox model analysis​ of impact of being homozygous for GPR65 rs3742704 polymorphism on overall survival in various patients’ groups (HR, Hazard Ratio), focused on grouped indications and individual indications with at least 2 samples for each genotype.

The partial GPR65 loss of function coding SNP rs3742704 associated with autoimmune and autoinflammatory disease susceptibility is present at relatively high frequencies in human populations (31, 32). We reasoned that stratifying cancer patients based on their GPR65 genotype may provide insights into the role of GPR65 in cancer immunity and we assessed patient overall survival (OS) as a function of GPR65 risk allele rs3742704 presence from the pan-cancer TCGA dataset. A Kaplan-Meier curve plot suggested a distinct survival pattern for rs3742704 homozygous patients compared with heterozygous patients or those bearing two copies of the GPR65 reference allele (**Supplementary Figure 1A**). Since the two latter groups showed near identical survival profiles and to gain statistical power, we performed a two-way analysis comparing rs3742704 homozygous patients against those bearing one or two copies of the reference GPR65 allele (referenced as non-homozygous patients or non-homozygotes afterwards). In this setting, the trend towards better survival for rs3742704 homozygotes was improved but did not reach statistical significance according to the logrank test or Cox proportional-hazards model on the pan-cancer dataset (**Figures 1A, C**). However, co-variate analysis focused on disease stage showed that rs3742704 homozygous patients diagnosed early (disease stage 1 or 2) showed significant survival benefit compared with non-homozygotes (**Figures 1B, C**). This did not hold for patients diagnosed at later stages (disease stage 3 or 4) (**Figures 1B, C**). Additional patients grouping based on their TME immune profile predicted by transcriptomics (37) suggested that, among patients with an inflammatory TME, rs3742704 homozygotes showed a trend towards better survival compared to non-homozygotes (**Supplementary Figure 1B**), whereas genotype stratification in other immune profile clusters showed no differences in survival.

### GPR65 mRNA expression profiles from public scRNAseq datasets

**Figure 2:**
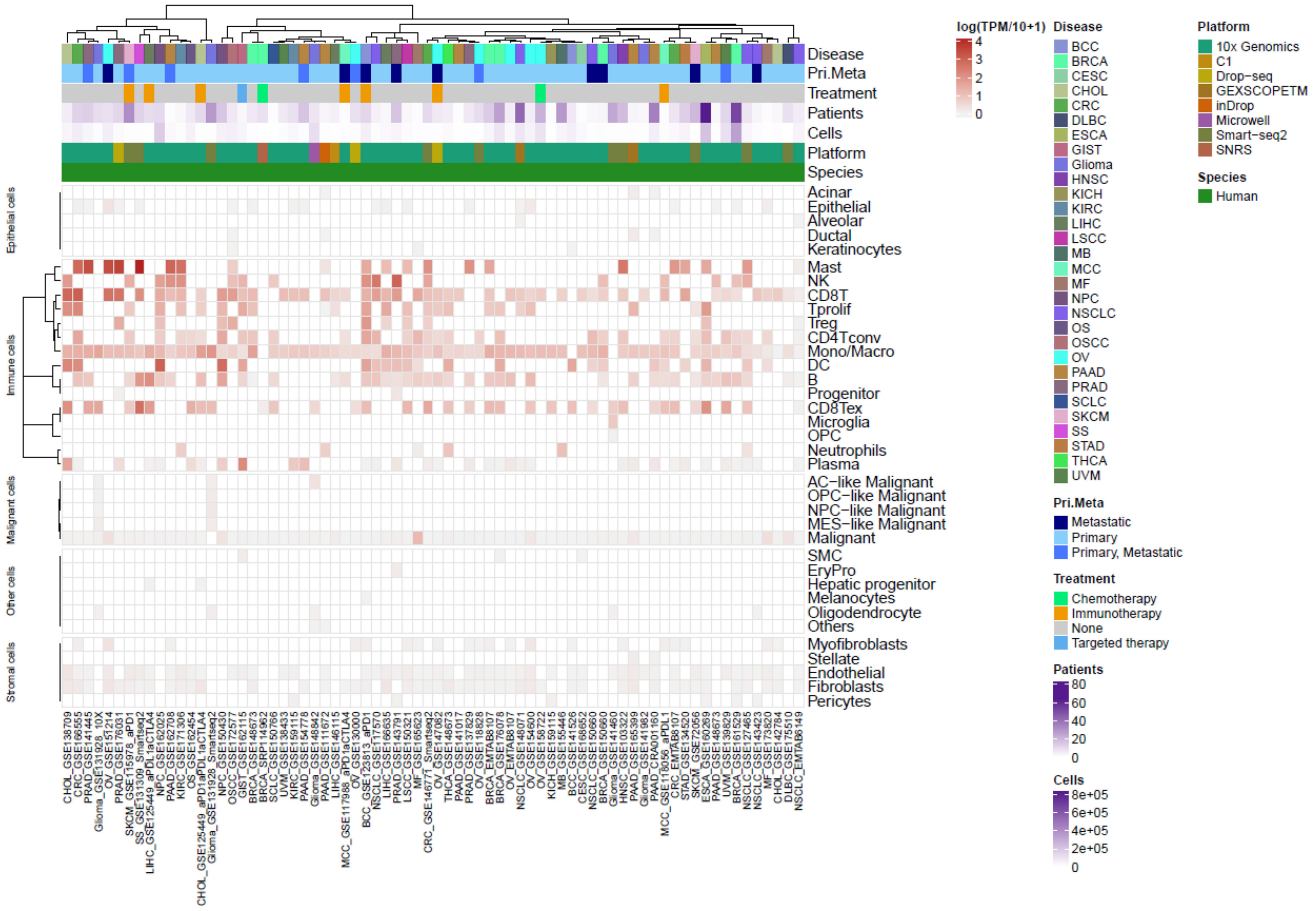
GPR65 mRNA expression profiling in cells of the tumour environment from the single cell RNAseq public resource TISCH2.

We collected from the TISCH2 resource (42) a total of 72 public TME scRNAseq datasets spanning 30 indications to assess GPR65 mRNA expression levels in tumour-infiltrating immune cells. A total of ~2.5M annotated cells from 36 distinct cell types, including 15 immune types, were analyzed. Consistent with previous reports, GPR65 gene expression was much higher in the immune compartment compared to non-immune cells in the TME of almost all indications (**Figure 2**). As previously reported, GPR65 gene expression was detected in almost all tumour-infiltrating monocytes/macrophages. In the same way, in almost all datasets, tumour-infiltrating CD8+ T-cells (CD8T) and exhausted CD8+ T-cells (exCD8T) expressed GPR65 gene. In addition, GPR65 gene expression levels were significantly higher in CD8T and exCD8T than in monocytes/macrophages cells (paired comparison in datasets where monocytes/macrophages and CD8T or exCD8T were annotated, p-value ~ 4E-3). B-cells, CD4+ T-cells (CD4T, excluding Tregs) and dendritic cells were the other cell types expressing GPR65 in most datasets in which they were annotated. These results establish that monocytes, macrophages and CD8+ T-cells were the most frequent GPR65 expressing tumour-infiltrating cells across a broad range of solid tumour indications.

To consolidate these findings, we explored BioTuring resource, a second pan-Cancer transcriptomic single cell database including ~98M annotated cells in over 1,600 studies and confirmed that GPR65 gene is frequently expressed by the previously identified cell populations. According to BioTuring annotations, 27.6% of CD8T, 28.2% of macrophages, 20.7% of tissue-resident macrophages and 18.8% of monocytes expressed GPR65 mRNA (**Supplementary Figure 2A**; **Supplementary Table 1**). Interestingly, other immune cell types such as mucosa associated invariant T-cells, intraepithelial lymphocytes, Th17 cells or γδ T-cells frequently expressed GPR65 at high levels, 58%, 51%, 36% and 32%, respectively, whereas fewer than 3% of malignant cells showed GPR65 gene expression. In terms of expression levels, GPR65 ranked within the 80^th^ percentile of genes most expressed by TILs (19% most highly expressed for CD8T cells and 20% for CD4T and B cells), and among the 71-76^th^ percentiles for monocytes/macrophages and 51^st^ percentile for malignant cells (**Supplementary Figure 2A**; **Supplementary Table 1**). We interrogated two datasets reporting single cell trancriptomics with cells isolated from tumour and non-tumour tissues (43, 44) to assess whether GPR65 expression was different in T-cells found infiltrating healthy, tumoural or allergic tissues. These data did not show any obvious GPR65 expression patterns matching tissue origin, indicating that GPR65 expression by T-cells seems homogenous for a particular organ, whether healthy or pathological, and is not enriched in cancer (**Supplementary Figures 2B, C**).

### GPR65 agonists inhibit the activation of antigen-specific human T-cells

**Figure 3:**
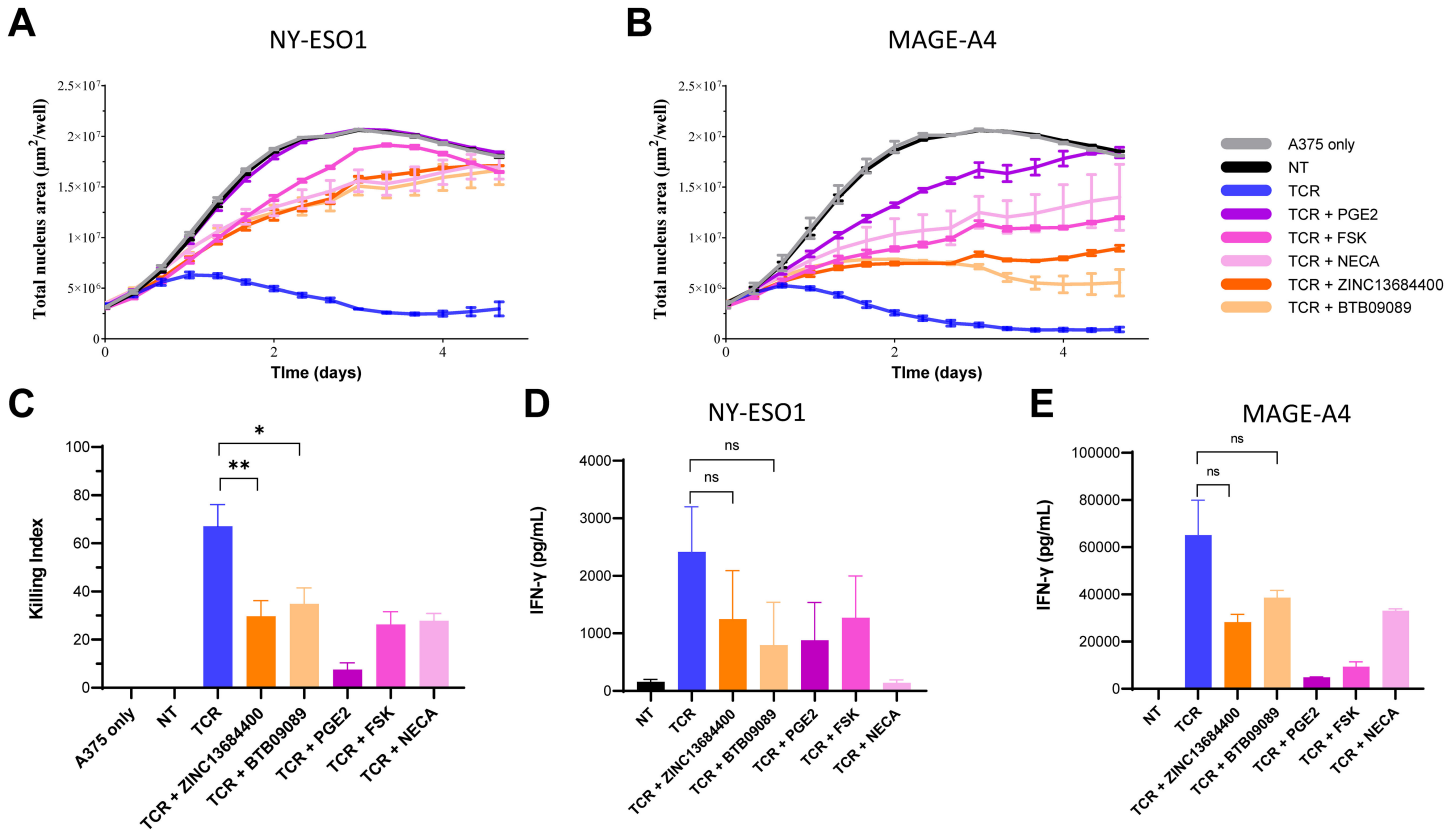
Suppression of human transgenic TCR T-cells effector functions by GPR65 agonists and cAMP elevating agents in response to antigen-specific activation by tumour cells. Representative examples of real-time fluorescent A375 cell growth inhibition induced by TCR T-cell cytotoxic activity in the HLA-A2/NY-ESO1 **(A)** and HLA-A2/MAGE-A4 **(B)** antigen systems. TCR T-cells and tumour cells were co-cultured at E:T = 5:1. The graph y-values represent A375 growth through the average total red nuclei surface area per well expressed in µm² from triplicates. The co-cultures were treated with DMSO vehicle control (TCR), 5µM ZINC13684400, 5µM BTB09089, 3 µM PGE2, 50 µM forskolin (FSK) or 33 µM NECA, as indicated in the figure key. NT = non-transduced T-cells control, TCR = T-cells transduced with the NY-ESO1 or MAGE-A4 specific TCRs. **(C)** Compilation of the T-cell killing activity for both TCR systems in each of the above conditions with T-cells from a total of five donors (two for the NY-ESO1 system and three for MAGE-A4) at E:T = 5:1. The killing index was calculated using area under the curve (AUC) values as follows: ((AUC of untreated A375-AUC of treated A375)/AUC of untreated A375) * 100. Inhibition of IFN-γ secretion in response to antigen by TCR T-cells expressing the NY-ESO1 **(D)** or MAGE-A4 TCRs **(E)** co-cultured with A375 cells at E:T ratio = 5:1; each assay condition was performed in duplicate. Unpaired t tests were used to assess the statistical significance of the differences between the groups compared as indicated (* p value = 0.0205; ** p value = 0.0098).

On the basis of GPR65 mRNA expression profiles, we investigated the effects of GPR65 agonism on T-cell effector functions. Two compounds described as GPR65 allosteric activators, BTB09089, a well-characterized and widely used GPR65 agonist, and ZINC13684400, were used in T-cell/tumour cell co-culture assays. Of note, both agonists had no effect on intra-cellular cAMP biosynthesis by A375 or on cell growth (**Supplementary Figure 3C**; **Supplementary Figure 4**). Total T-cells engineered to express transgenic TCRs from lentiviral vectors specific for the tumour associated epitopes HLA-A2/NY-ESO1_157-165_ or HLA-A2/MAGE-A4_230-239_, both expressed by the A375 target cell line, were exposed to GPR65 agonists in killing and cytokine secretion assays. In both antigen systems, the GPR65 agonists significantly inhibited T-cell cytotoxic activity against target cells (**Figures 3A–C**) compared to vehicle control conditions. In addition, IFN-γ secretion was also reduced when T-cells were co-cultured with A375 cells in presence of BTB09089 and ZINC13684400 (**Figures 3D, E**), although this inhibition did not reach statistical significance. The extent of T-cell effector functions suppression by the GPR65 agonists was comparable with other mediators inhibiting T-cell activation through Gαs-coupled GPCRs, since high concentrations of both PGE2 and the adenosine receptor agonist NECA had similar effects on killing and IFN-γ secretion (**Figures 3A–E**). In addition, the adenylate cyclase activator forskolin (FSK), which elevates intra-cellular cAMP levels independently of any cell-surface receptor, also efficiently suppressed transgenic TCR T-cells in the same co-culture assays. These results indicate that GPR65 agonism efficiently inhibits T-cell activation and effector functions.

### Validation of GPR65 small molecule inhibitors blocking pH-dependent cAMP induction

**Figure 4:**
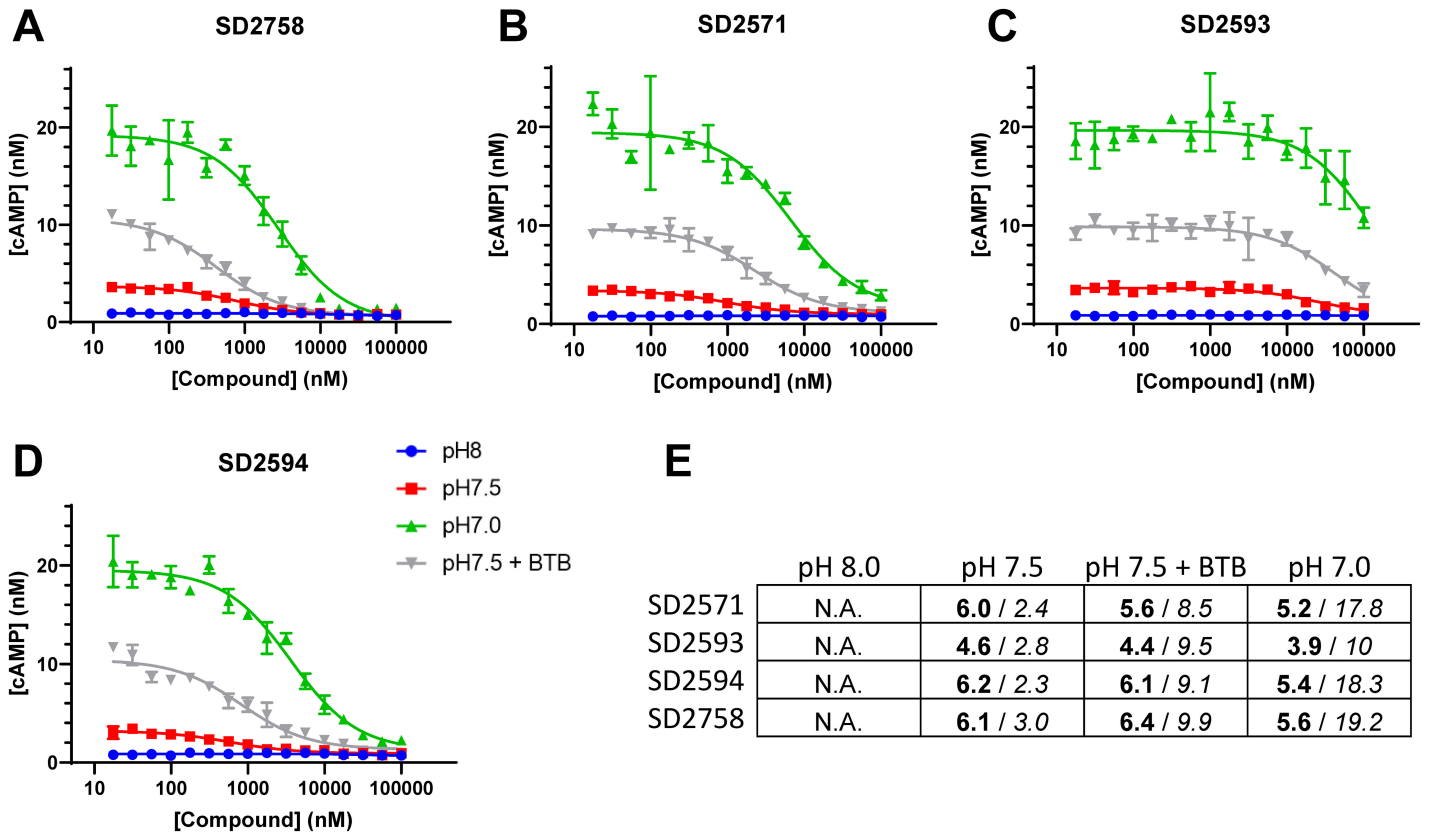
Identification and characterization of GPR65 small molecule inhibitory compounds. **(A–D)** Dose-response curves showing intra-cellular cAMP levels as a function of GPR65 antagonists, concentrations measured at 3 different extra-cellular pH or with 1µM of the GPR65 agonist BTB09089 at pH 7.5. Potencies, expressed as pIC_50_ values (in bold), and amplitudes, expressed as maximal difference in concentration (nM) of cAMP (in *italic*) of several antagonists were evaluated to assess sensitivity of the screening assay at different pH values and in the presence of the agonist BTB09089 (BTB) **(E)**.

In order to further probe the functions of GPR65 in T-cells, we sought to validate and use inhibitory small molecule compounds described as GPR65 antagonists (patent international application PCT/GB2021/051397). Four compounds were synthesized (**Supplementary Figure 5**) and assessed for their capacity to inhibit cAMP biosynthesis upon GPR65 activation by BTB09089 or by increases in extra-cellular H+ concentrations. A preliminary de-activation step, performed by incubating GPR65-overexpressing A375 cells at pH8, was required to reduce basal intra-cellular cAMP levels. Subsequent exposure of the cells to decreasing pH resulted in GPR65-dependent increases in cAMP that peaked at pH7 (data not shown). Out of the four tested GPR65 inhibitors, three fully blocked cAMP induction at pH 7.5 and all but one (SD2593) also partially blocked cAMP induction at pH 7 in a dose-dependent manner (**Figures 4A–D**). The two most potent compounds SD2594 and SD2758 displayed sub-micromolar IC_50_ values at pH 7.5 and between 1 and 10 µM at pH 7 (**Figure 4E**). Additionally, the same four compounds prevented cAMP induction in response to BTB09089 with efficiencies and potencies comparable to those observed at pH 7.5 (**Figures 4A–E**). These experiments thus validated several small molecules antagonizing the effects of GPR65 activating ligands, including H+, and provided us with tool compounds to characterize GPR65 inhibition in functional T-cell assays.

### GPR65 inhibition augments the cytotoxic activity of antigen-specific human T-cells

**Figure 5:**
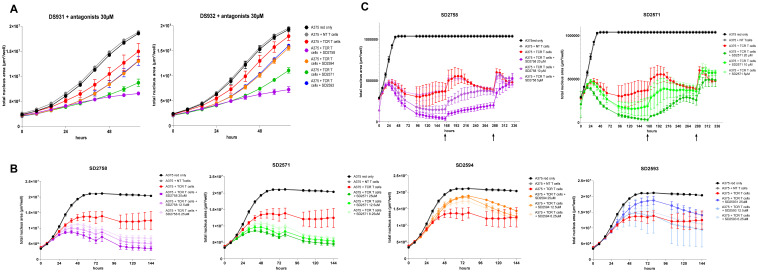
GPR65 inhibitors increase T-cell antigen-specific tumour cell killing. **(A)** NY-ESO1 TCR T cells from 2 healthy donors (generated as described in Material and Methods, DS931 and DS932) were co-cultured with red A375 cells and treated or not with selected GPR65 antagonists at 30 µM and at a 5:1 E:T cell ratio. **(B)** NY-ESO1 TCR T cells from DS932 were thawed and co-cultured with red A375 cells and treated or not with selected GPR65 antagonists at 6.25, 12.5 and 25 µM and at a 10:1 E:T cell ratio. **(C)** NY-ESO1 TCR T cells from a healthy donor (DS673) were co-cultured with red A375 cells and treated or not with selected GPR65 antagonists at different concentrations, as indicated, and at a 10:1 E:T cell ratio. Growth of tumour cells was followed over time by Incucyte through red fluorescence and depicted as area of live cells. N.B. that growth of tumour cells only and tumour cells + non transduced T cells is maintained at the same value from 48h due to biological variabilities. Raw data can be found in **Supplementary Figure 6C**. In total TCR-T cells from 8 donors were tested for this assay. DS931 and DS932 were used in three independent experiments and 6 additional donors were used in 2 independent experiments. NT, non transduced.

The four validated antagonists were assessed in *in vitro* T-cell functional assays with the HLA-A2/NY-ESO1 system. TCR-T cells co-cultured with A375 cells showed efficient tumour cell killing, which was further improved in presence of the GPR65 inhibitors, SD2758 and SD2571 being the most active compounds (**Figure 5A**; **Supplementary Figures 6B**, **C**). Interestingly, in HEPES-free culture conditions, we observed a higher killing from TCR-T cells independently of the antagonists, which dampened the detection window to evaluate killing activity by GPR65 inhibition (**Supplementary Figure 7**). The assay was repeated on one of the same donors, and the effect of the two inhibitors SD2758 and SD2571 was confirmed in a dose-response fashion (6.25-12.5-25 µM) (**Figure 5B**). We observed no clear response with SD2593 and SD2594, combined with a higher standard deviation observed over time (**Figure 5B**). We next addressed the serial killing capabilities of TCR-T cells treated with the GPR65 antagonists, focusing on SD2758 and SD2571 only. Co-culture was performed with a slightly different concentration range of the inhibitors to capture the suboptimal effect (5-10-20 µM) and TCR T-cells were rechallenged with A375 cells on days 7 and 12 in presence of both compounds. We confirmed the dose-response activity of SD2758 and SD2571 with maintenance of the improved killing capability over 10 days (**Figure 5C**; **Supplementary Figure 6C**). Of note, inhibitory effects of the SD compounds are partially lost at 5 µM in these donors.

### GPR65 inhibition leads higher T-cell proinflammatory response

**Figure 6:**
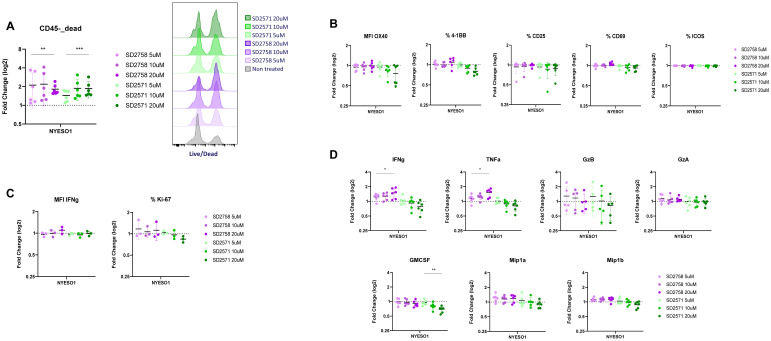
GPR65 inhibition is associated with a higher production of pro-inflammatory cytokines. **(A)** NY-ESO1 TCR T cells from 6 healthy donors (n=2 independent experiments) were co-cultured with wild type A375 cells and treated or not with selected GPR65 antagonists at different concentrations for 3 days. Frequencies of live cells among total CD45- cells were analyzed by FACS and fold change was calculated for each condition compared to the non-treated one. One-way ANOVA test was performed on frequencies of CD45-live cells (left panel). Histograms of the L/D staining in one representative donor (right panel). **(B)** FACS analysis was performed as described in **(A)** and frequencies or MFI expression of T-cell activation markers were assessed. Fold change was calculated for each treatment condition compared to the non-treated one. **(C)** In the second assay assessing T-cell activation, an intranuclear staining was performed following surface staining to analyze in addition IFN-γ and Ki-67 expression. **(D)** Supernatants from the same co-cultures were harvested at day 3 and analyzed for cytokine release using Luminex (n=6, 2 independent experiments, technical replicates=3). One-way ANOVA test was performed on cytokine concentrations.

As our tumour-antigen killing system showed a GPR65 antagonist-driven increased T cell cytotoxicity, we assessed any correlation with T cell functional phenotypes by assessing TCR-driven T cell activation markers, proliferation and intracellular IFN-γ production. Upon three days of cell co-culture in presence of the compounds, we harvested cells for FACS staining and supernatants for cytokine release analysis. Although flow cytometry seems to be less sensitive in detecting a dose-response effect of the antagonists, we observed an increase in dead CD45- cells frequencies in treated versus non-treated samples, at the three doses tested for both SD2758 and SD2571 (**Figure 6A**). This was further confirmed by unaffected frequencies of live TCR-T cells in different treatment conditions (**Supplementary Figures 8A, B**). When gated on TCR+ T cells, no difference in activation markers (OX40, 4-1BB, CD25, CD69 nor ICOS) was observed between cells treated or not with the antagonists (**Figure 6B**). This was also the case for proliferation (Ki-67) or intracellular IFN-γ production (**Figure 6C**). We then measured the concentrations of cytokines secreted in the co-culture supernatants, focusing on well-described proinflammatory molecules (IFN-γ, TNF-α, Granzyme B, Granzyme A, GMCSF, Mip-1α and Mip-1β), and detected significant higher production of IFN-γ and TNF-α in samples treated with SD2758 (**Figure 6D**). These results indicate that the promoted T-cell killing activity by GPR65 inhibition is associated with an increased pro-inflammatory cytokine production.

## Discussion

In this study, we explored the potential of GPR65 as a target for cancer immunotherapy and its relevance to human T-cell biology. Our computational studies showed that a GPR65 loss of function SNP linked to autoimmunity correlated with better patient survival in the TCGA database, and that TILs isolated from a range of solid tumours expressed GPR65 mRNA transcripts at levels similar or superior to myeloid cells, known to express functional GPR65 proteins. Based on these findings, we studied primary human T-cells and showed that GPR65 agonism inhibited the functions of T-cells stimulated with tumour-associated antigens. Furthermore, we validated novel GPR65 small compound antagonists that promoted the anti-tumour activity of T-cells and increased their cytokine secretion. Collectively, these results consolidate previous research reporting that GPR65 may be a therapeutically relevant target in IO and expand the range of its immune-suppressive activity to T-cells, which represent a major effector cell population in cancer immunotherapy.

Our GPR65 genotype stratification analyses revealed an association between homozygosity for the I231L GPR65 loss of function variant (rs3742704) and an improved survival across all TCGA cancer types (**Figure 1A**). Interestingly, covariate analyses pointed to a significant beneficial survival association for patients diagnosed early (disease stage 1 or 2), whereas patients bearing the same homozygous variant allele but diagnosed at stage 3 or 4 showed no survival benefit (**Figure 1B**). This observation is difficult to interpret in a highly heterogenous cohort such as TCGA but could suggest that patients bearing the loss of function GPR65 allele may better benefit from early treatment lines. Further fractioning of the patient population based on tumour immune contexture hinted that the positive correlation between survival and the GPR65 SNP may be more pronounced in the context of an inflammatory TME (**Supplementary Figure 1B**). These results expand on recently communicated *in silico* studies documenting the link between the GPR65 variant and improved cancer patient survival (45) and suggest that pharmacological GPR65 inhibition could better benefit specific therapeutic settings.

Next, we queried public scRNAseq datasets to determine the relative expression levels of GPR65 mRNA in immune cells of the TME across multiple cancer indications. Based on standard TISCH cell type annotations, it appeared that tumour-infiltrating CD8+ T-cells expressed high GPR65 mRNA levels comparable to monocytes and macrophages, known to express functional amounts of GPR65 protein. Moreover, significant GPR65 mRNA expression was also observed in “CD8+ T-cells” and “exhausted CD8+ T-cells” in the datasets where the considered populations were annotated (**Figure 2**). Interestingly, a recent report highlighted GPR65 as one of the most highly expressed GPCRs in terminally exhausted patients’ CD8+ T-cells, along with well-known G-αs coupled GPCRs associated with T-cell dysfunction such as the adenosine receptor A2A or the prostaglandin receptors EP2 and EP4 (16). Our own analyses do not allow to directly confirm this differential GPR65 expression within these CD8+ T-cell subsets because sample heterogeneity across all datasets preclude such comparisons. Nevertheless, our results point to substantial GPR65 expression in tumour CD8+ T-cells relative to other cell types and, by extension, reinforce the notion that GPR65 is expressed at functionally relevant levels in CD8+ T-cells, including the dysfunctional/exhausted subsets. Next, we sought to substantiate these observations by querying datasets from the Bioturing resource, which confirmed frequent GPR65 mRNA expression within the 80^th^ percentile of genes expressed by CD8+ T-cells (**Supplementary Figure 2**; **Supplementary Table 1**). These expression levels and frequencies were slightly higher for CD8+ T-cells than for monocytes and macrophages, thereby corroborating our initial findings in the TISCH database. Of note, all the commercial antibodies we tested lacked reactivity or specificity towards the GPR65 protein in flow cytometry and immuno-histochemistry studies, thus preventing us from validating cellular GPR65 expression at the protein level (**Supplementary Table 3**).

Using validated agonistic small molecule compounds, we showed that GPR65 stimulation resulted in the efficient inhibition of T-cell effector functions upon activation by tumour cells bearing natural levels of peptide/HLA antigens, similar to what T-cells may encounter in the context of an immune response to a patient tumour. Both target cell killing and cytokine secretion activities of total human T-cells redirected with a lentiviral vector against two peptide/HLA-I epitopes were efficiently inhibited by the GPR65 agonists BTB095089 and ZINC13684400. The extent of this inhibition was comparable to PGE2 and the non-selective adenosine receptor agonist NECA, two stimuli relevant to cancer immune suppression, as well as Forskolin, which all efficiently restrain T-cell signaling through the cAMP/PKA pathway. These results imply that GPR65 elicits direct T-cell inhibition signals resulting in inefficient killing and cytokine release in conditions where the receptor is efficiently agonized. Since H+ concentrations are locally increased in several solid tumours, it is conceivable that GPR65 acts to dampen the anti-cancer activity of T-cells present in the TME.

To gain further insights into the biology of GPR65 in T-cells, we used pharmacological agents recently described as GPR65 antagonists (46). We assessed and validated the antagonistic activity of several compounds and selected two based on selectivity and potency against a synthetic GPR65 agonist as well as against H+, the natural GPR65 agonist. These GPR65 antagonists efficiently decreased cAMP levels at pH 7, indicating that, in cells overexpressing GPR65 at least, neutral levels of H+ efficiently agonize GPR65. Indeed, maximum GPR65 agonism was reached at pH 7 in our experimental system, which is in keeping with previous reports (10, 39). Moreover, T-cells co-cultured with target tumour cells in standard cell culture medium showed improved effector functions in presence of both GPR65 antagonists, implying that GPR65 likely restrains T-cell functions in non-acidic conditions. These antagonists improved tumour antigen-specific killing by T-cells without showing direct cytotoxic effects on tumour cells, strongly suggesting that blocking GPR65 signaling enhanced T-cell killing in response to antigen. Moreover, the increase in T-cell cytokine secretion in co-culture assays or upon antibody-mediated stimuli also supports a direct effect of GPR65 antagonists on T-cell functions, likely by dampening the inhibitory effects of protons present in the culture medium. These results imply that GPR65 suppresses T-cell functions in non-acidic milieux, and it follows that pharmacologically blocking GPR65 in a therapeutic setting would likely enhance T-cell functions at neutral and acidic pH, where maximal GPR65 signaling is triggered. Of note, the TCR T-cells used in our assays were generated with clinically relevant affinity-enhanced TCRs using a production process comparable to most autologous cancer T-cell therapies. Our results therefore imply that blocking GPR65 signaling in CAR and TCR T-cells through pharmacological or genetic approaches could increase their therapeutic efficacy.

The GPR65 antagonist tool compounds characterized in our study may prove valuable to further investigate the functions of GPR65. However, although we unequivocally demonstrated that SD2758 and SD2571 efficiently inhibited GPR65 signaling, we cannot completely rule out off-target effects and additional validation of these compounds is pending to fully establish their potential as agents highly selective for GPR65. The fact that both compounds increased T-cell cytotoxic activity yet only SD2758 promoted cytokine secretion is also intriguing and may underpin different potencies or mechanisms of action between these two compounds. Genetic loss of function studies, such as CRISPR/Cas9 knock-out approaches, would best address potential compound selectivity issues and complement our work using GPR65 small molecule inhibitors.

Our findings provide several lines of evidence in support of a suppressive role for GPR65 in T-cell responses against cancer. Notably, we show that GPR65 agonism, like other G-α coupled GPCRs, substantially altered human T-cell activation and functions in response to tumour cells. However, our experimental models have limitations and did not enable us to accurately assess the effects of defined H+ concentrations on T-cell functions since controlling the cell culture medium pH over several days in dynamic co-culture systems is exceedingly challenging. Nevertheless, our results suggest that T-cell inhibition by GPR65 takes place in neutral to slightly acidic pH, i.e., in conditions relevant to multiple organs and milieux, and not only in restricted acidic niches. This implies that GPR65 may exert broad immune-regulatory functions on T-cells and other immune cells at steady state. Consequently, pharmacological modulation of GPR65 could benefit a broad range of pathologies, such as cancer and autoimmunity, where immune modulation is desirable. Consolidation of this hypothesis will require extensive validation at the preclinical stage and in clinical settings as there are numerous open questions on the role of GPR65 in immunity in general and on T-cell biology in particular. For instance, the respective roles of GPR65 on CD4+ and CD8+ T-cells was not addressed in our study and remain unclear. Additionally, GPR65 inhibition was shown to skew the differentiation of CD4+ T-cells towards a Th1 profile at the expense of Th17 polarization (33), which could be beneficial in the context of most anti-tumour immune responses. Single cell transcriptomic comparisons between T-cells isolated from non-tumoural and tumoural gastric or lung tissues showed similar levels of GPR65 mRNA, indicating that GPR65 expression does not seem to be increased at cancer sites (**Supplementary Figures 2B, C**). Therefore, pharmacological modulation of GPR65 would likely affect all T-cells in a similarly without selectivity towards TILs.

Altogether, the work reported in this article lays the foundation for further investigations into the role of GPR65 as an immune-suppressive agent of the TME and in its potential as a target for immunotherapies aiming to harness the anti-tumoural activity of immune cells, including T-cells, in solid tumours. An upcoming clinical trial in cancer with the small molecule GPR65 inhibitor PTT-4256 was recently announced and may provide clues as to the therapeutic potential of GPR65 modulation.

## Data Availability

The raw data supporting the conclusions of this article will be made available by the authors, without undue reservation.
