# Supplementary figures and images for: In silico and pharmacological evaluation of GPR65 as a cancer immunotherapy target regulating T-cell functions
Source: Front Immunol. 2024 Oct 17;15:1483258. doi: 10.3389/fimmu.2024.1483258 (PMC11525786; doi:10.3389/fimmu.2024.1483258)

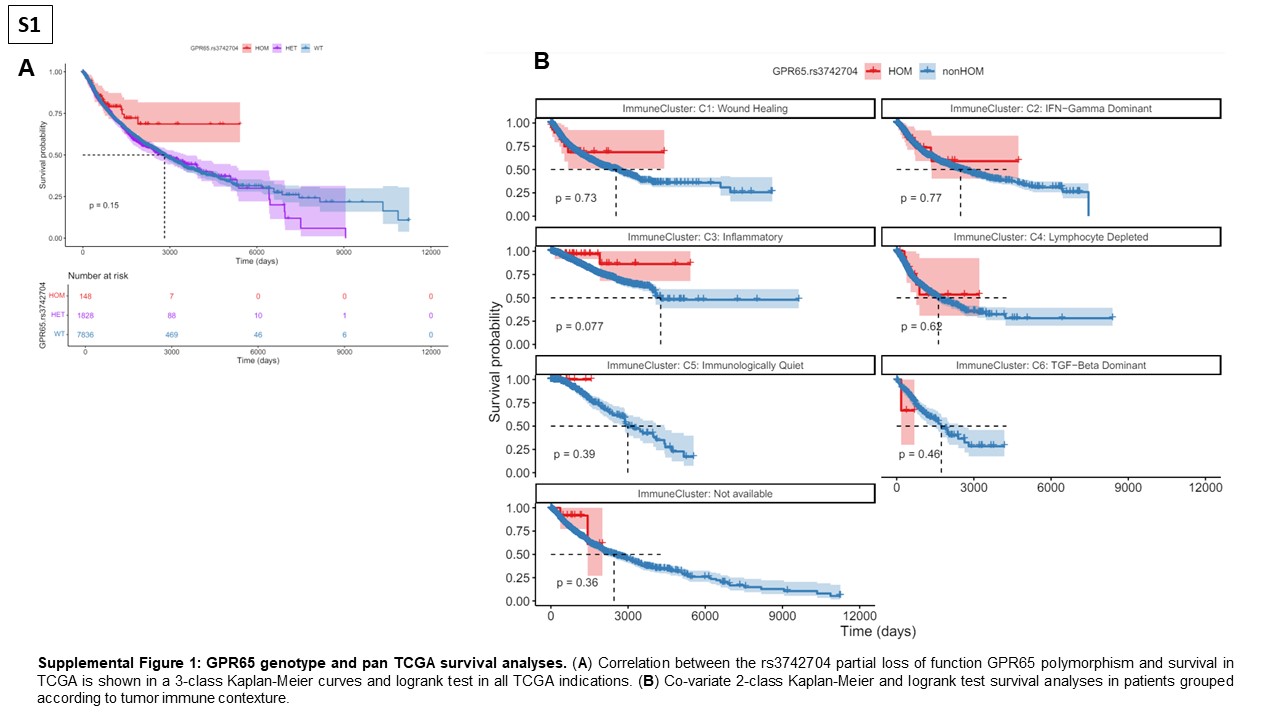

Supplement: Supplementary file 1 [file Image1.jpeg]

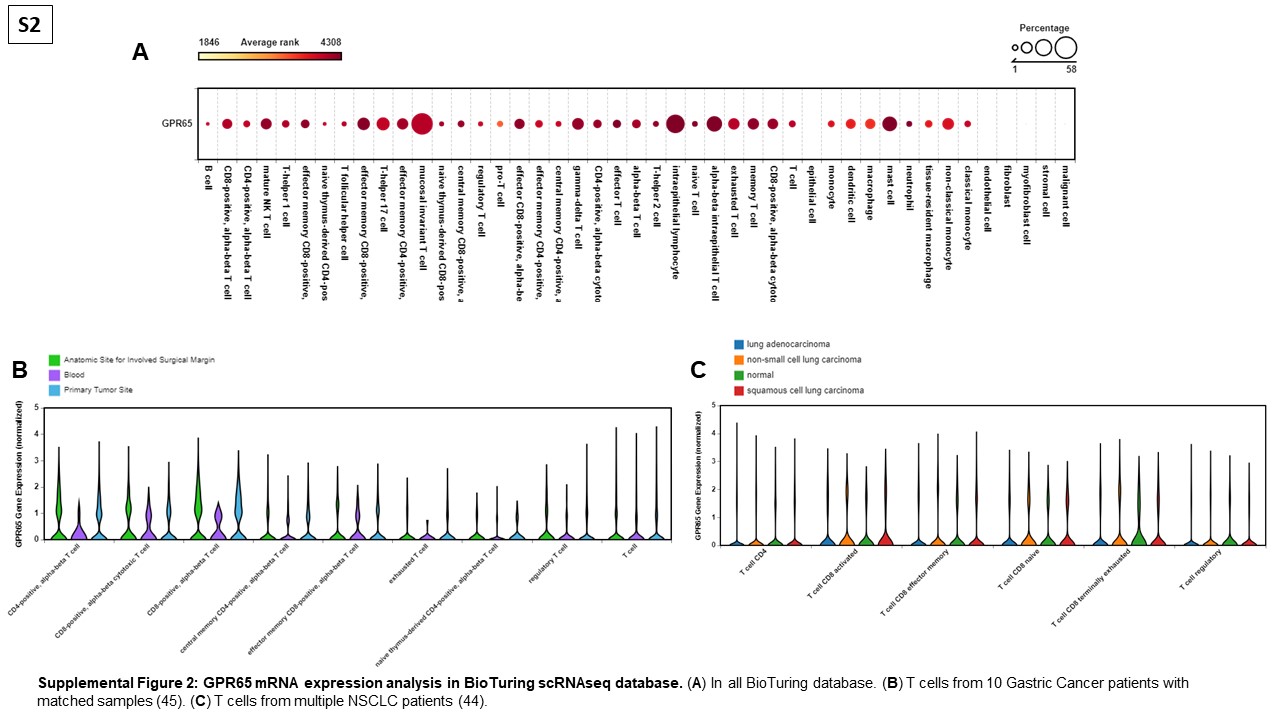

Supplement: Supplementary file 2 [file Image2.jpeg]

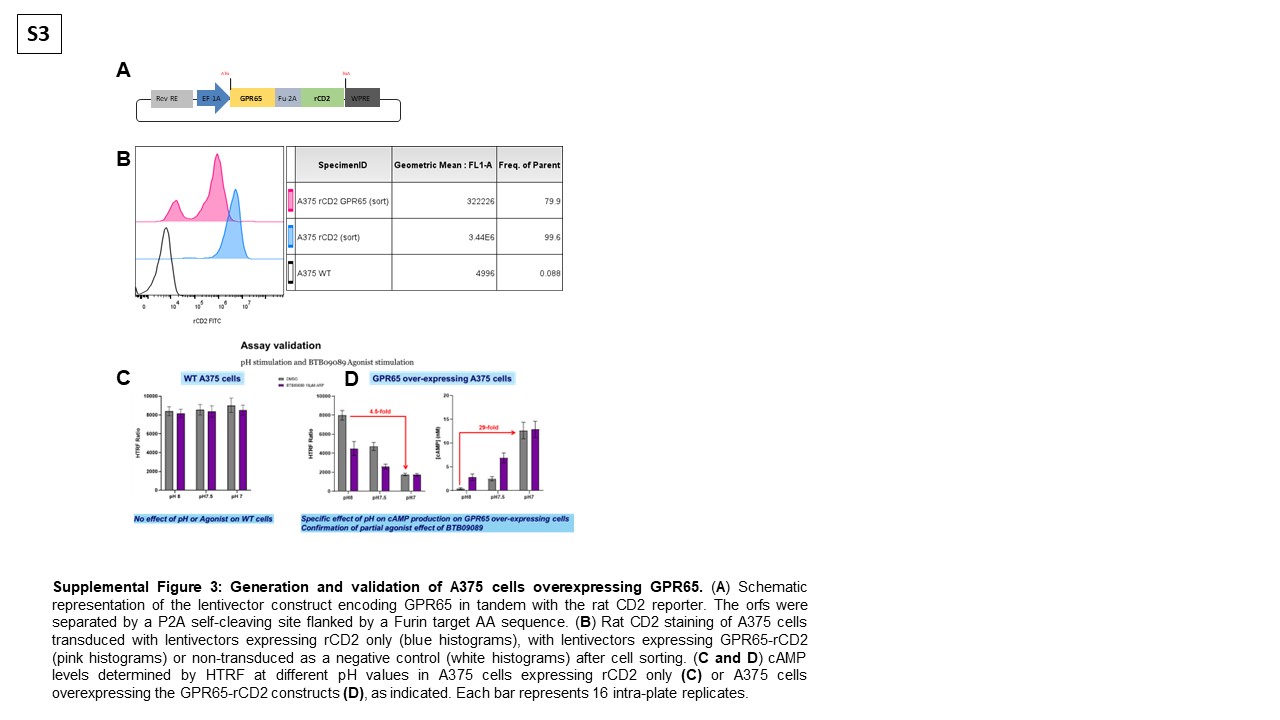

Supplement: Supplementary file 3 [file Image3.jpeg]

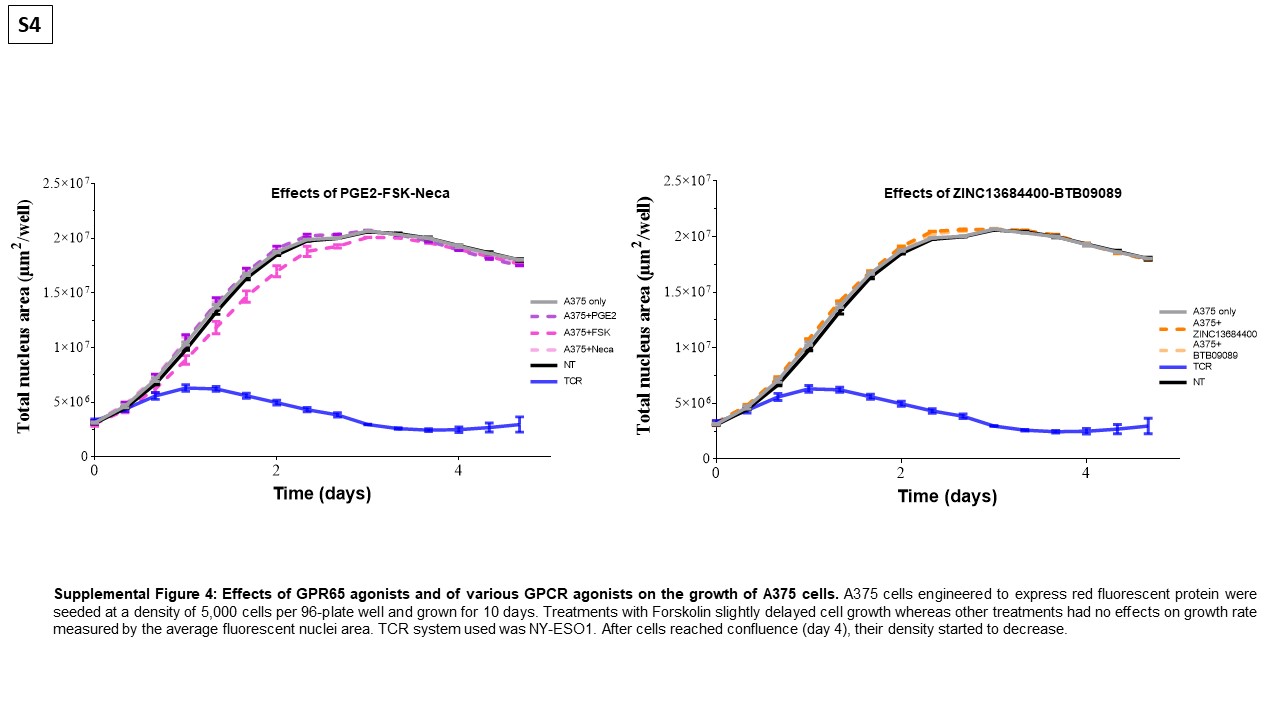

Supplement: Supplementary file 4 [file Image4.jpeg]

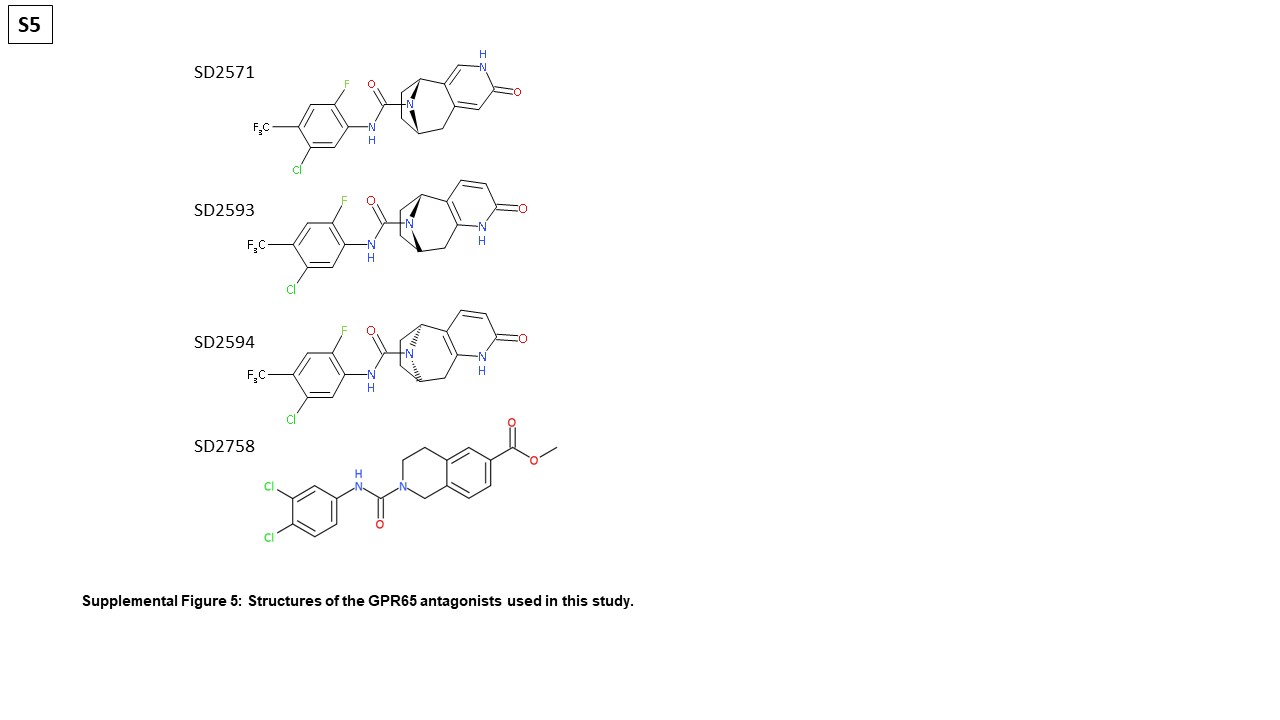

Supplement: Supplementary file 5 [file Image5.jpeg]

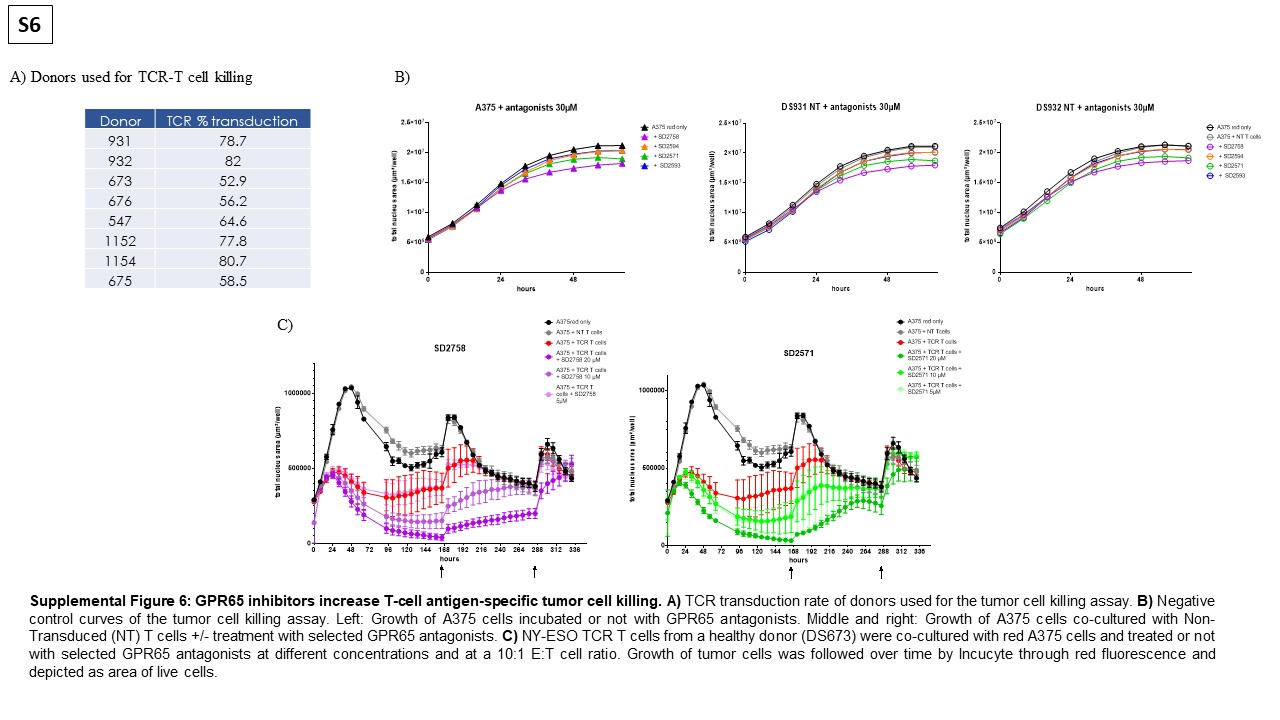

Supplement: Supplementary file 6 [file Image6.jpeg]

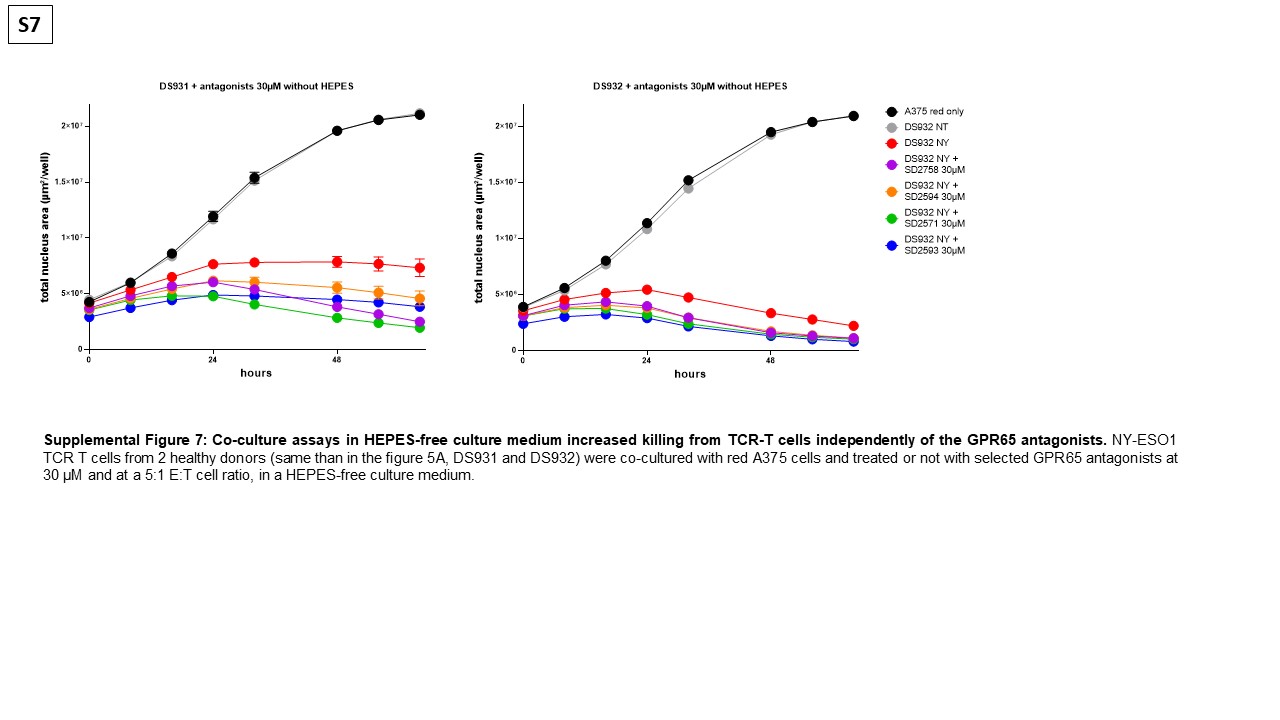

Supplement: Supplementary file 7 [file Image7.jpeg]

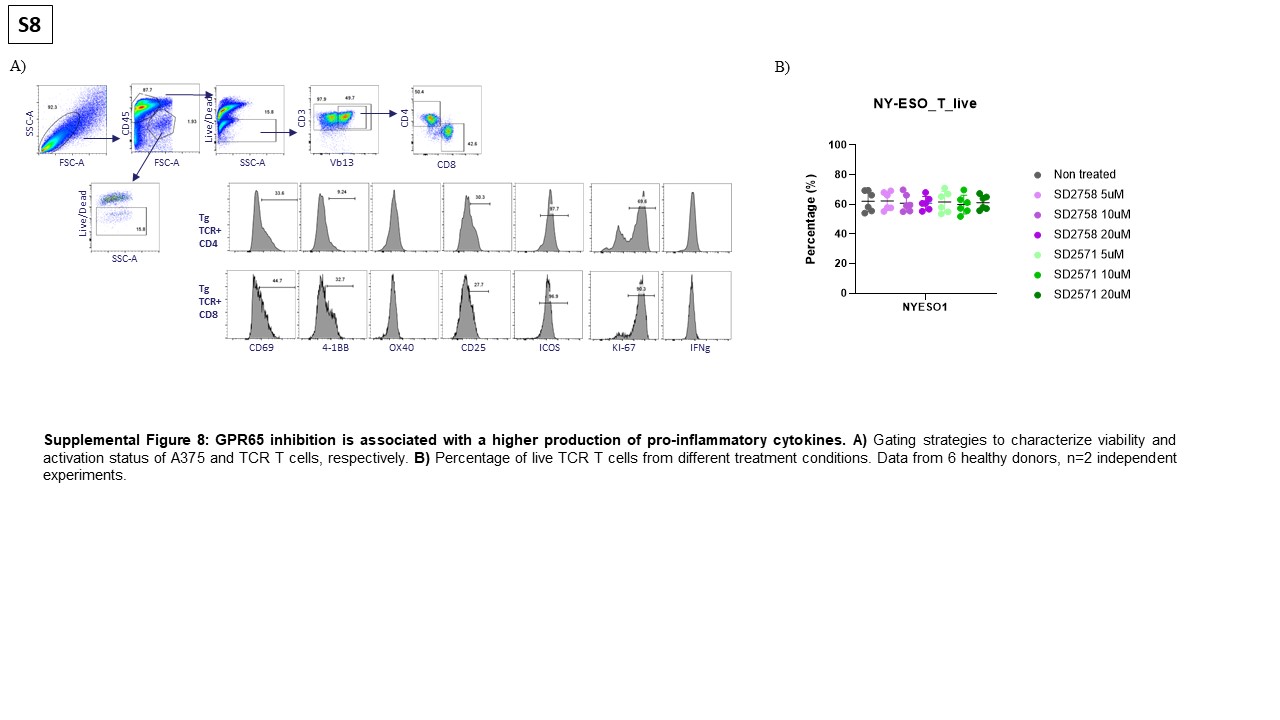

Supplement: Supplementary file 8 [file Image8.jpeg]

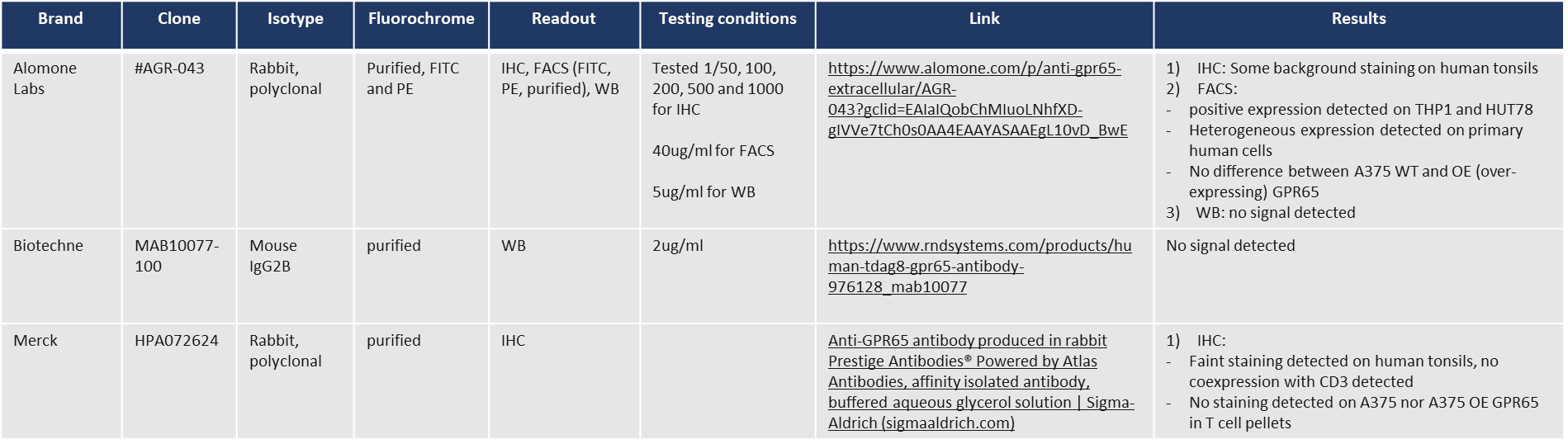


Supplementary Table 3. Information on tested GPR65 antibodies

Supplement: Supplementary file 11 [file Table3.docx]
