# Supplementary material for: In silico and pharmacological evaluation of GPR65 as a cancer immunotherapy target regulating T-cell functions
Source: Front Immunol. 2024 Oct 17;15:1483258. doi: 10.3389/fimmu.2024.1483258 (PMC11525786; doi:10.3389/fimmu.2024.1483258)
Supplement: Supplementary file 9 [file Table1.docx]

| **Cell type** | **Total number of cells** | | **Percentage of GPR65 gene expressing cells** | | **Number of GPR65 gene expressing cells** | | **Average rank of GPR65 as a top expressed gene** | **Average percentile of GPR65 as a top expressed gene** |
| --- | --- | --- | --- | --- | --- | --- | --- | --- |
| **mucosal invariant T cell** | 479633 | 58.40 | | 280103 | | 4030 | | 80.58 |
| **intraepithelial lymphocyte** | 15838 | 50.52 | | 8001 | | 4346 | | 86.9 |
| **alpha-beta intraepithelial T cell** | 8932 | 42.17 | | 3767 | | 4304 | | 86.06 |
| **mast cell** | 218629 | 40.28 | | 88064 | | 4463 | | 89.24 |
| **T-helper 17 cell** | 139577 | 35.74 | | 49888 | | 3979 | | 79.56 |
| **effector memory CD8-positive, alpha-beta T cell, terminally differentiated** | 156231 | 34.24 | | 53487 | | 4274 | | 85.46 |
| **gamma-delta T cell** | 373472 | 32.18 | | 120175 | | 4209 | | 84.16 |
| **non-classical monocyte** | 589215 | 32.07 | | 188963 | | 3804 | | 76.06 |
| **exhausted T cell** | 337704 | 31.23 | | 105464 | | 4024 | | 80.46 |
| **memory T cell** | 205277 | 31.11 | | 63858 | | 4229 | | 84.56 |
| **effector memory CD4-positive, alpha-beta T cell, terminally differentiated** | 20211 | 30.86 | | 6238 | | 4126 | | 82.5 |
| **mature NK T cell** | 259187 | 30.22 | | 78316 | | 4211 | | 84.2 |
| **CD8-positive, alpha-beta cytotoxic T cell** | 303023 | 29.22 | | 88534 | | 4181 | | 83.6 |
| **effector CD8-positive, alpha-beta T cell** | 568959 | 28.20 | | 160440 | | 4230 | | 84.58 |
| **macrophage** | 2489382 | 28.18 | | 701615 | | 3568 | | 71.34 |
| **CD8-positive, alpha-beta T cell** | 3874165 | 27.59 | | 1068882 | | 4061 | | 81.2 |
| **dendritic cell** | 352746 | 27.22 | | 96005 | | 3669 | | 73.36 |
| **alpha-beta T cell** | 52144 | 24.11 | | 12572 | | 4123 | | 82.44 |
| **effector memory CD8-positive, alpha-beta T cell** | 970563 | 23.92 | | 232153 | | 4234 | | 84.66 |
| **CD4-positive, alpha-beta cytotoxic T cell** | 106716 | 23.29 | | 24859 | | 4175 | | 83.48 |
| **effector T cell** | 832239 | 22.77 | | 189478 | | 4368 | | 87.34 |
| **effector memory CD4-positive, alpha-beta T cell** | 464689 | 21.05 | | 97824 | | 3923 | | 78.44 |
| **T-helper 1 cell** | 119701 | 20.77 | | 24860 | | 4069 | | 81.36 |
| **tissue-resident macrophage** | 214984 | 20.69 | | 44476 | | 3714 | | 74.26 |
| **T cell** | 2523941 | 19.18 | | 484011 | | 4026 | | 80.5 |
| **CD4-positive, alpha-beta T cell** | 4209640 | 19.07 | | 802790 | | 3983 | | 79.64 |
| **monocyte** | 3305587 | 18.83 | | 622358 | | 3792 | | 75.82 |
| **central memory CD8-positive, alpha-beta T cell** | 192687 | 18.73 | | 36093 | | 4189 | | 83.76 |
| **Cell type** | **Total number of cells** | **Percentage of GPR65 gene expressing cells** | | **Number of GPR65 gene expressing cells** | | **Average rank of GPR65 as a top expressed gene** | | **Average percentile of GPR65 as a top expressed gene** |
| **classical monocyte** | 1308082 | 18.20 | | 238030 | | 3954 | | 79.06 |
| **pro-T cell** | 13988 | 17.25 | | 2413 | | 3317 | | 66.32 |
| **T-helper 2 cell** | 89184 | 16.53 | | 14738 | | 4215 | | 84.28 |
| **neutrophil** | 832543 | 16.15 | | 134489 | | 4587 | | 91.72 |
| **central memory CD4-positive, alpha-beta T cell** | 1323059 | 15.93 | | 210767 | | 3945 | | 78.88 |
| **naive T cell** | 160359 | 15.69 | | 25155 | | 4327 | | 86.52 |
| **regulatory T cell** | 955457 | 14.01 | | 133836 | | 3917 | | 78.32 |
| **T follicular helper cell** | 355830 | 13.96 | | 49662 | | 4043 | | 80.84 |
| **naive thymus-derived CD8-positive, alpha-beta T cell** | 1084649 | 13.82 | | 149912 | | 4136 | | 82.7 |
| **naive thymus-derived CD4-positive, alpha-beta T cell** | 3415747 | 10.56 | | 360692 | | 3975 | | 79.48 |
| **B cell** | 5138427 | 9.98 | | 512918 | | 3979 | | 79.56 |
| **malignant cell** | 1627317 | 2.83 | | 46067 | | 2567 | | 51.32 |
| **myofibroblast cell** | 176182 | 1.65 | | 2902 | | 3177 | | 63.52 |
| **fibroblast** | 3545420 | 0.69 | | 24437 | | 3358 | | 67.14 |
| **endothelial cell** | 2950697 | 0.59 | | 17283 | | 3508 | | 70.14 |
| **stromal cell** | 742632 | 0.55 | | 4062 | | 3415 | | 68.28 |
| **epithelial cell** | 6692644 | 0.51 | | 33971 | | 3156 | | 63.1 |

Supplementary Table 1. Details of BioTuring scRNAseq data extracted from the database for analyses.
