# Supplementary material for: In silico and pharmacological evaluation of GPR65 as a cancer immunotherapy target regulating T-cell functions
Source: Front Immunol. 2024 Oct 17;15:1483258. doi: 10.3389/fimmu.2024.1483258 (PMC11525786; doi:10.3389/fimmu.2024.1483258)
Supplement: Supplementary file 10 [file Table2.docx]

| **Dye** | **Antibody** | **Supplier** | **Cat #** | **Clone** |
| --- | --- | --- | --- | --- |
| Aqua | Zombie | Biolegend | 423101 |  |
| APC-Vio770 | CD4 | Miltenyi | 130-113-211 | VIT4 |
| Vioblue | CD8 | Miltenyi | 130-110-683 | REA734 |
| BUV496 | CD4 | BD Biosciences | 612936 | SK3 |
| BV421 | 4-1BB | Biolegend | 309820 | 4B4-1 |
| BV785 | CD25 | Biolegend | 302638 | BC96 |
| FITC | CD69 | BD Biosciences | 555530 | FN50 |
| PerCP | CD8 | Biolegend | 344708 | SK1 |
| PE | TCR Vβ13.1 (NYESO1) | Beckman coulter | IM2292 | IMMU 222 |
| PE-Dazzle594 | OX40 | Biolegend | 350020 | Ber-ACT35 (ACT35) |
| PE-VIO770 | CD45 | Miltenyi | 130-113-119 | 5B1 |
| AF700 | ICOS | Biolegend | 313528 | C398.4A |
| APC-Cy7 | CD3 | Biolegend | 300318 | HIT3a |
| BV605 | IFNg | BD Biosciences | 562974 | B27 |
| AF647 | KI-67 | BD Biosciences | 561126 | B56 |

Supplementary Table 2: Antibodies used for flow cytometry analysis.
